# A qualitative study on the post-discharge self-management experiences and needs of patients with secondary lymphedema

**DOI:** 10.1097/MD.0000000000043557

**Published:** 2025-08-15

**Authors:** Yinyin Guan, Hui Ju, Lanwei Xu, Zhenzhen Wan, Lei Ge, Yuxia Fang

**Affiliations:** a Department of Hand, Foot and Micro Reconstruction Surgery, Shandong Provincial Hospital Affiliated to Shandong First Medical University, Jinan, Shandong Province, China; b Department of Hand, Foot and Micro Reconstruction Surgery, Shandong Provincial Hospital, Cheeloo College of Medicine, Shandong University, Jinan, Shandong Province, China; c Department of Emergency, People’s Hospital of Rizhao, Jining Medical University, Shandong Province, China.

**Keywords:** chronic disease trajectory framework, phenomenological research, secondary lymphedema, self-care

## Abstract

Guided by the Chronic Illness Trajectory Framework, this study aimed to explore the post-discharge self-management experiences of patients with secondary lymphedema following lymphovenous anastomosis surgery, providing evidence for developing targeted interventions. A descriptive phenomenological design was employed. Through purposive sampling, 16 participants who underwent lymphovenous anastomosis surgery were recruited in February 2025. Semi-structured interviews were conducted and analyzed using Colaizzi phenomenological analysis method. Within the dimensional framework of the Chronic Illness Trajectory Model, 7 main themes emerged regarding patients’ home-based self-management experiences: inadequate knowledge of postoperative lymphedema rehabilitation, improper self-management practices, barriers to accessing disease management information, challenges in daily life, recognition of and hope for social support, insufficient capacity to manage internal stress, and acceptance of the disease and self-reconciliation. Patients after lymphovenous anastomosis surgery experience multidimensional challenges including informational gaps, functional impairments, and psychosocial adaptation difficulties. A trajectory-oriented care model integrating stepped education, adaptive symptom management, and multidisciplinary support is recommended.

## 1. Introduction

Secondary lymphedema is caused by non-primary factors leading to lymphatic system damage or obstruction, resulting in impaired lymphatic fluid drainage and subsequent interstitial fluid accumulation and swelling in the affected area.^[1]^ In some patients with malignant tumors, lymph node dissection and radiation therapy often disrupt lymphatic pathways, leading to complications such as swelling, fat deposition, tissue fibrosis, infections, and deformities in the affected limb,^[2]^ which significantly impact both physical and mental health. Lymphovenous anastomosis (LVA) is a microsurgical procedure for treating lymphedema by anastomosing lymphatic vessels with veins, allowing lymphatic fluid to reenter the venous system and effectively alleviating lymphedema.^[3]^ Postoperative LVA patients still require long-term home-based self-management, including compression therapy, manual lymphatic drainage, rehabilitation exercises, skin care, weight control, and psychological support.^[4]^ Research indicates that the quality of self-management is crucial for maintaining therapeutic effects and enhancing patients’ self-care abilities, as well as slowing disease progression, which can significantly improve the quality of life of patients with chronic diseases.^[5–8]^ Currently, most studies have focused on the diagnosis, prevention, surgery, and perioperative care of lymphedema.^[9,10]^ There is a lack of attention to home care following LVA surgery, which is specifically manifested in the inadequate understanding of the difficulties patients face during the rehabilitation process and their need for personalized nursing guidance.^[11,12]^ As a result, patients’ self-management at home is ineffective, leading to recurrent conditions, increased psychological burden, and waste of medical resources. It also hinders healthcare providers from developing effective care plans and affects the overall improvement of treatment levels.

This study focuses on exploring the self-management experiences of patients with secondary lymphedema following LVA surgery. By employing qualitative research methods, we overcome the limitations of quantitative research in terms of preset variables and research tools. This approach allows us to flexibly capture important yet hard-to-quantify information, such as patients’ cognitive biases about the disease, subtle psychological adjustments, and genuine feelings about family and social support, which are crucial for developing precise intervention measures. Based on the Chronic Illness Trajectory Framework,^[13,14]^ we conducted a comprehensive analysis of patients’ postoperative home-based self-management status, experiences, and needs from 3 core dimensions: illness-related behaviors (patients’ understanding of the disease and management measures), self-concept behaviors (individuals’ perception of self-worth and self-maintenance), and everyday-life behaviors (internal and external behaviors such as emotional management, stress coping, and role adaptation). By integrating qualitative data with the theoretical framework, this study can deeply reveal patients’ behavioral patterns, psychological changes, and social interactions during the self-management process. This lays a solid theoretical foundation for developing postoperative home-based self-management strategies that meet patients’ actual needs.

## 2. Materials and methods

### 2.1. Study design

This qualitative study employed descriptive methods.^[15]^ Ideally, researchers engage in direct communication and interaction with participants. Through in-depth conversations, the researchers explored the participants’ thought processes and their subjective experiences from various perspectives. The data and information gathered were then thoroughly summarized and organized. From February to March 2025, we utilized qualitative research techniques to investigate the home self-management experiences of patients with secondary lymphedema following lymphovenous anastomosis surgery.^[16]^ We adhered to the EQUATOR’s COREQ checklist to ensure the quality of this qualitative study.^[17]^

### 2.2. Participants and recruitment

The study population consisted of patients with secondary lymphedema who underwent LVA surgery at the Shandong First Medical University Affiliated Shandong Provincial Hospital.

Inclusion criteria: (1) age ≥ 18 years; (2) a history of malignant tumor and related treatments, with unilateral limb swelling; (3) underwent LVA surgery and had been home-rehabilitating for over 1 month after discharge; (4) clear consciousness and normal communication and comprehension abilities; (5) provided informed consent and voluntarily participated in the interviews. Exclusion criteria: patients with severe heart, liver, or kidney diseases.

Purposeful sampling was employed in this study. This study employs the principle of saturation in qualitative research as proposed by Saunders B.^[18]^ Researchers determined whether participants met the inclusion and exclusion criteria through the medical records system. They contacted the patients who met the inclusion criteria, inquired whether they were willing to participate in the study, and provided them with written information for their consideration. In this study, as researchers collected information through interviews, observations, and other methods, the frequency of new information or new themes gradually decreased until no new information or themes emerged. Meanwhile, the research team repeatedly verified the identified themes and information and held team discussions. The research team believed that sufficient data had been collected to comprehensively and in-depth answer the research questions. Under these circumstances, the researchers considered the data to be saturated and ceased further data collection. A total of 16 participants (coded P1–P16) were interviewed.

### 2.3. Research methods

#### 2.3.1. Development of the interview guide

In accordance with the research objectives and the 3 core dimensions of the chronic illness trajectory model (illness-related behaviors, self-concept behaviors, and daily life behaviors),^[13,14]^ and after consulting with experts in lymphedema specialty medical and nursing care, physical therapists, and psychologists, an initial interview guide was developed. Three patients were selected for pilot interviews, and the guide was revised based on the results of these interviews. The final interview guide included the following questions:

① How much do you know about self-management at home after LVA surgery?
② How have you managed your condition after discharge?
③ What difficulties in daily life and work have you encountered during the process of self-management at home after surgery?
④ What support have your family and the outside world provided during your self-management at home after surgery? What kind of help do you expect to receive?
⑤ How has your mindset changed from the time of surgery to the period of self-management at home?

#### 2.3.2. Data collection methods

This study was conducted in Jinan, Shandong Province, China. Face-to-face semi-structured interviews were carried out by nursing master’s students Ju Hui and Fang Yuxia, who had received training in qualitative research. Ju Hui conducted the interviews in the participants’ homes, while Fang Yuxia did so in a quiet office at the hospital to ensure a comfortable and private environment. Before the interviews, the research purpose and confidentiality principles were explained to the participants, and written informed consent was obtained. Demographic information such as age and gender was recorded, and the interview content was anonymized to protect privacy. With the participants’ consent, the interviews were recorded and transcribed verbatim into Chinese. Techniques such as questioning, repetition, and summarization were used, and additional information was supplemented by handwritten notes taken on-site. The interviews lasted between 30 and 60 minutes. During the study, there were no cases of participants withdrawing or undergoing repeat interviews.

#### 2.3.3. Data organization and analysis

Two researchers used Nvivo12 software and the Colaizzi 7-step analysis method^[19]^ to analyze the collected data, employing iterative induction and collaborative team coding methods to code and analyze the text.^[20]^ Initially, the researchers transcribed the audio data and carefully read the interview records to immerse themselves and gain an overall understanding. Subsequently, we extracted significant statements sentence by sentence, coded recurring and meaningful viewpoints, and developed a coding manual. We then aggregated the coded viewpoints to form preliminary thematic clusters. We defined and described these themes, identified similar viewpoints, and refined the thematic concepts. Finally, we verified the findings with the participants to ensure the authenticity and reliability of the study. In addition, when disagreements arose during the data analysis process, the research team held 2 meetings to thoroughly review the raw data, continuously compare and categorize the information, discuss among themselves, and review and modify the coding manual and existing thematic framework. All codes in the coding manual were applied to the 16 transcribed texts, which were then refined, simplified, and expanded.

#### 2.3.4. Quality control

To ensure the credibility of this study, we implemented several measures. Prior to the interviews, we established a trusting relationship with the patients. During the interviews, the researchers avoided expressing personal opinions or evaluating the patients’ viewpoints, suspending their own feelings and perspectives as much as possible and adjusting the order of the interview guide according to the actual situation of the respondents. After the interviews, the researchers promptly followed up with the patients on key issues and provided a brief summary of the interview highlights, allowing the patients or their family members the opportunity to supplement or correct the summary. We also employed triangulation by comparing interview notes with the actual transcribed content to verify the reliability of the data sources. Additionally, the concurrent data collection and iterative analysis in this study served as a validation method, enhancing the credibility of the research. Moreover, collecting demographic and clinical characteristics of the participants also provided support for generalizing the study results to other populations.

#### 2.3.5. Ethical approval

This study was reviewed and approved by the Ethics Review Committee of the Shandong First Medical University Affiliated Shandong Provincial Hospital (No.: SWYX: No. 2025-049). Meanwhile, our study adhered to the Helsinki Declaration. All participants provided written consent to take part in the feasibility trial and were re-consented by a member of the research team prior to participating in the qualitative interviews.

## 3. Results

A total of 16 patients with secondary lymphedema were included in this study as participants, and their sociodemographic characteristics are detailed in Table 1. In terms of gender, all participants were female. The age range was from 34 to 79 years old, covering different age groups. The main causes of the disease were lymphedema resulting from surgeries for cervical cancer and breast cancer, with disease duration varying from 8 months to 19 years. The primary methods of medical payment were urban employee and urban-rural resident basic medical insurance. The educational levels were widely distributed, ranging from primary school to university. All participants were married, and their occupations were diverse, including farmers, workers, civil servants, freelancers, clerks, and retirees.

**Table 1:** General information of respondents (n = 16).

No.	Gender	Age	Etiology and duration of illness	Medical payment method	Education level	Marital status	Occupation
1	Female	35	Lymphedema in left lower limb after cervical cancer surgery for 2 years	Urban Resident Basic Medical Insurance	Junior High School	Married	Farmer
2	Female	54	Lymphedema in left lower limb after cervical cancer surgery for 3 years	Urban Resident Basic Medical Insurance	Primary School	Married	Farmer
3	Female	34	Lymphedema in right upper limb after breast cancer surgery for 2 years	Urban Employee Basic Medical Insurance	High School	Married	Worker (Commercial Service)
4	Female	49	Lymphedema in left lower limb after cervical cancer surgery for 5 years	Urban Resident Basic Medical Insurance	Primary School	Married	Farmer
5	Female	46	Lymphedema in left upper limb after breast cancer surgery for 5 years	Urban Resident Basic Medical Insurance	Junior High School	Married	Unemployed
6	Female	67	Lymphedema in both lower limbs after cervical cancer surgery for 19 years	Urban Employee Basic Medical Insurance	Junior High School	Married	Worker (Retired)
7	Female	56	Lymphedema in left upper limb after breast cancer surgery for 11 years	Urban Resident Basic Medical Insurance	Primary School	Married	Freelancer
8	Female	49	Lymphedema in right upper limb after breast cancer surgery for 13 years	Urban Employee Basic Medical Insurance	University	Married	Civil Servant
9	Female	61	Lymphedema in right upper limb after breast cancer surgery for 4 years	Urban Employee Basic Medical Insurance	Junior High School	Married	Worker (Retired)
10	Female	50	Lymphedema in left upper limb after breast cancer surgery for 1 year	Urban Employee Basic Medical Insurance	High School	Married	Freelancer
11	Female	62	Lymphedema in left upper limb after breast cancer surgery for 1 year	Urban Resident Basic Medical Insurance	Junior High School	Married	Unemployed
12	Female	45	Lymphedema in left upper limb after breast cancer surgery for 5 years	Urban Employee Basic Medical Insurance	Junior High School	Married	Freelancer
13	Female	69	Lymphedema in left lower limb after cervical cancer surgery for 7 years	Urban Resident Basic Medical Insurance	Primary School	Married	Farmer
14	Female	79	Lymphedema in left lower limb after cervical cancer surgery for 8 months	Urban Employee Basic Medical Insurance	Junior High School	Married	Worker (Retired)
15	Female	46	Lymphedema in left lower limb after left lower limb trauma surgery for 4 years	Urban Employee Basic Medical Insurance	University	Married	Clerk
16	Female	50	Lymphedema in left upper limb after breast cancer surgery for 1 year	Urban Employee Basic Medical Insurance	High School	Married	Freelancer

Based on the dimensions of illness-related behaviors, self-concept behaviors, and daily living behaviors in the chronic illness trajectory model, the interview data from 16 participants were repeatedly organized, coded, categorized, and summarized. This process ultimately generated 58 initial codes, from which 7 themes and 15 categories related to the family self-management experiences of patients with secondary lymphedema were identified. Table 2 is an example of the data coding analysis.

**Table 2:** Example of data coding analysis.

Theme	Categories	Initial codes
Insufficient Knowledge of Postoperative Rehabilitation for Lymphedema	Insufficient Self-Management Knowledge	Confusion of rehabilitation priorities at different stages (e.g., engaging in high-intensity upper limb exercises in the early postoperative period/ still excessively restricting activities in the late stage).
		Lack of emphasis (e.g., “I didn’t finish watching the video on manual drainage, and I didn’t take it seriously”).
		Cognitive biases (e.g., “The doctor said I can’t do heavy work, so I usually avoid physical activity”).
	Discrepancy between Self-Management Behavior and Knowledge	Conflicts with lifestyle (e.g., prolonged sitting due to work).
		Insufficient continuity in self-management (e.g., frequently interrupting the use of compression sleeves due to social activities).
	Neglect of Condition Monitoring and Follow-up	Failure to follow medical advice for follow-up (e.g., “I didn’t go for a follow-up and don’t want to, it’s useless”).
		Failure to follow medical advice for monitoring (e.g., “I can tell the leg is swollen just by looking, no need to measure the circumference”).
	Insufficient Experience in Managing Complications	Inadequate ability to identify and respond to complications (e.g., not seeking medical attention promptly when experiencing skin erythema or blisters, applying unknown medications instead).
		Pessimistic expectations for managing complications (e.g., “Infections will keep recurring, treatment is useless”).
Barriers in Obtaining Disease Management Information	Limited Access and Ability to Obtain Information	Inability to use digital platforms proficiently (e.g., not knowing how to use WeChat mini-programs).
		Lack of family support (family caregivers lack rehabilitation knowledge).
		Poor accessibility of medical resources (few authoritative postoperative self-management knowledge available on WeChat or short videos).
	Low Willingness to Obtain Information Due to Psychological Factors	Low self-efficacy leading to a fear of difficulty (e.g., believing one cannot learn compression treatment methods, thus avoiding information).
Inappropriate Self-Management	Insufficient Mastery of Rehabilitation Skills	Improper control of the tension and density of low-elasticity bandage wrapping.
		Arbitrary and inconsistent execution of manual drainage (abandoned after learning from short videos due to poor results).
		Monotonous and non-standardized functional exercises (e.g., only performing simple movements like “raising and lowering the arm”).
		Delayed or omitted skin care (forgetting to apply moisturizing lotion until skin dryness occurs).
		Non-standardized methods for measuring edema at home (using non-medical soft tape measures or inconsistent measurement sites).
	Insufficient Support for Management Resources	Lack of proper guidance and supervision (no rehabilitation therapist providing home visits).
		Incomplete follow-up mechanisms by medical staff (e.g., phone communication alone is insufficient to correct operational details).
	Poor Adherence	Insufficient duration of wearing compression devices
		Loss of control in dietary management.
		Resistance to using doctor-recommended lymphatic drainage techniques, air pressure therapy devices, etc.

### 3.1. Disease-related behavioral dimension

#### 3.1.1. Theme 1: insufficient postoperative rehabilitation awareness

##### 3.1.1.1. Categorie 1: lack of awareness of self-management

In the interviews, some patients had an incomplete understanding of self-management knowledge, and during home care, they paid insufficient attention to the self-management measures for lymphedema in the affected limb, resulting in many misconceptions.

Inadequate knowledge of postoperative rehabilitation for lymphedema. “What is functional exercise, does walking count?” [Participant 2, 3-year lymphedema]; “I only know about bandaging and compression treatment, I do it every day.”[Participant 9, 4-year lymphedema]; “Do I need to control my diet? I don’t eat spicy food, but I haven’t paid attention to anything else.”[Participant 10, 1-year lymphedema].

Insufficient emphasis on postoperative rehabilitation for lymphedema. “In the early stage after discharge, I did manual lymphatic drainage twice a day, but I stopped later. I felt it wasn’t very useful, just a little massage.” [Participant 4, 5-year lymphedema]; “The doctor sent me a rehabilitation exercise video, but I take care of my 2 grandsons at home, so I don’t have time to do exercises!” [Participant 7, 11-year lymphedema]; P11: “I didn’t even finish watching a manual lymphatic drainage video, I didn’t take it seriously.” [Participant 11, 1-year lymphedema].

Misconceptions about postoperative rehabilitation for lymphedema. “Bandaging might help a little, but I don’t think other things are that meaningful.”[Participant 2, 3-year lymphedema]; “I think manual lymphatic drainage is less effective than bandaging the leg, so I didn’t do the manual drainage.” [Participant 6, 19-year lymphedema]; “The doctor said not to do heavy work at home, so I don’t really do much, just take it slow.” [Participant 2, 8-month lymphedema].

##### 3.1.1.2. Categorie 2: behavioral and cognitive contradictions in self-management

Some participants were aware that their disease management was inadequate, but due to long-term personal habits and social customs, they still intermittently relaxed their management. “Sometimes I just half-heartedly do manual lymphatic drainage. I’m already tired from picking up the kids and doing some work.” [Participant 1, 2-year lymphedema]; “The doctor told me to lose weight. I lost 2 pounds right after I was discharged, but I couldn’t keep it up. When I’m hungry, I just eat. I’m like that, I eat whatever I want (laughs).” [Participant 9, 4-year lymphedema]; “I know manual lymphatic drainage is important, but when I’m tired, I just don’t want to do anything.”[Participant 11, 1-year lymphedema].

##### 3.1.1.3. Categorie 3: neglecting disease monitoring and follow-up

Some patients, due to busy family affairs and a lack of concern for their condition, neglected monitoring disease indicators and symptoms. “I never measured the circumference of my leg because I could clearly see that 1 leg is thick, and the other is thin.”[Participant 1, 2-year lymphedema]; “I’m helping take care of my 2 sons’ children, so it’s not very convenient to measure my arm.” [Participant 11, 1-year lymphedema]; “I don’t go for follow-ups, there’s no point. It’s just teaching how to exercise and do drainage, there’s nothing else to do.” [Participant 15, 4-year lymphedema].

##### 3.1.1.4. Categorie 4: lack of experience in managing complications

Patients generally lack experience in the prevention and early treatment of complications, mainly reflected in insufficient awareness of complications and a lack of effective prevention and handling measures. “Once my incision area became red, itchy, and had lumps, I hadn’t worn the bandage for several days. The iodine tincture from the clinic didn’t work. In the end, I went to the pharmacy and bought some anti-inflammatory medicine, and that worked.” [Participant 1, 2-year lymphedema]; “This summer, it was so hot, and my arm got inflamed from being covered up. I had a fever for several days before going to the hospital. The doctor said my arm was infected, and I had to get injections for more than ten days before it was better.” [Participant 9, 4-year lymphedema].

#### 3.1.2. Theme 2: barriers to obtaining disease management information

Accessing disease-related information through various online channels helps patients adopt effective self-care behaviors [18]. According to patients, primary healthcare institutions and community health service centers have relatively low awareness of lymphedema, which makes it difficult for patients to obtain effective treatment and rehabilitation support. Some patients also mentioned that they can find information about lymphedema rehabilitation treatment through WeChat, short videos, etc, but there is still relatively little knowledge about self-management. “I went to the local hospital to change the dressing and remove the stitches, and they didn’t know about this disease. The community doesn’t know either. When I want to ask something, I feel very helpless.” [Participant 2, 3-year lymphedema]; “There are videos online about swelling treatment, but there’s very little about what to pay attention to after surgery. It would be great if there were popular science videos or WeChat public accounts on this, so we could learn more about post-surgery rehabilitation.” [Participant 8, 13-year lymphedema]; “I don’t really trust the information online; I don’t know which ones are authoritative. Can you send out a brochure or something? That way we’d feel more assured reading it.” [Participant 9, 4-year lymphedema].

#### 3.1.3. Theme 3: suboptimal self-management practices

##### 3.1.3.1. Categorie 1: compression bandaging treatment

“Bandaging looks simple, but it’s really not that easy when doing it. Sometimes the bandage is too tight or the pressure is off, and my whole hand turns purple, numb, and painful. If I don’t do it right, my hand swells up a lot by the end of the day.”[Participant 3, 2-year lymphedema]; “Sometimes the bandage isn’t even, leaving marks that take several days to go away.” P12: “After bandaging, my ankle became more swollen. The doctor said I did it wrong, but it’s really hard to get the pressure right.” [Participant 8, 13-year lymphedema]; “Bandaging at home isn’t as standard as in the hospital. I often forget this or that, and many times I didn’t follow the healthcare staff’s instructions. Later, I just hurriedly bandaged it however I could.” [Participant 13, 7-year lymphedema].

##### 3.1.3.2. Categorie 2: manual lymphatic drainage

“Doing housework takes a lot of time and energy. I only do manual lymphatic drainage when I remember, and when I forget, I just skip it.” [Participant 1, 2-year lymphedema]; “I learned manual lymphatic drainage from TikTok, but later I felt it wasn’t very useful, so I stopped doing it.” [Participant 2, 3-year lymphedema]; “The doctor sent me a manual lymphatic drainage video on my phone. I followed it for 7 or 8 minutes, but sometimes I stop halfway through.”[Participant 13, 7-year lymphedema].

##### 3.1.3.3. Categorie 3: functional exercise

Most patients mechanically followed the doctor’s advice, and their understanding and actions regarding functional exercise remained superficial. “Right after discharge, I did a few sets of rehabilitation exercises, but I didn’t keep it up over time.” [Participant 4, 5-year lymphedema]; P9: “My usual rehabilitation exercise is just lifting my arm high and moving it around.” [Participant 9, 4-year lymphedema]; “I wore the bandage for a long time, and after removing it, I didn’t really move my hand much, so in the end, my hand couldn’t even bend.” [Participant 10, 1-year lymphedema]; “I’m afraid to exercise my swollen arm. The only exercise I do is just lifting it up and down like that for rehabilitation.” [Participant 11, 1-year lymphedema].

##### 3.1.3.4. Categorie 4: skin care

Proper skin care after surgery is crucial for accelerating the healing of the LVA surgery incision and preventing infection, while also helping to identify complications early. However, some patients neglected the importance of this daily care. “Sometimes I forget to apply lotion. When I’m busy, I also skip it to save time.” [Participant 2, 3-year lymphedema]; “At first, I completely forgot about it. It wasn’t until I noticed my skin cracking 1 day that I remembered the doctor told me to do skin care.”[Participant 13, 7-year lymphedema].

### 3.2. Daily life behavior dimension

#### 3.2.1. Theme 1: daily life disturbances

Most participants reported that while pressure therapy helped alleviate their condition, it also caused various inconveniences in daily life, such as restricted limb movement, discomfort from tightness, changes in urination habits, and impaired sleep quality. “Wearing the bandage feels very restrictive. I can’t bend my leg properly when riding an electric bike, and going up and down stairs or going out is inconvenient.” [Participant 1, 2-year lymphedema]; “When doing housework or looking after the children, the bandage keeps slipping down every time I squat and stand up. It’s really annoying.” [Participant 2, 3-year lymphedema]. “After putting on the bandage, I feel like I need to urinate more frequently. Each time, it’s not much, but I keep feeling the urge to go.” [Participant 4, 5-year lymphedema]; “I can’t sleep well at night because I’m always worried about pressing on my arm and making the swelling worse. I don’t dare to press on it or touch it, and it’s not as comfortable as before.” [Participant 5, 5-year lymphedema]. “I can’t tolerate the pressure from the bandage. Even doing a little bit of work makes me uncomfortable and sore. It feels so restrictive, but once I take it off, I feel much better.” [Participant 8, 13-year lymphedema]; “Wearing compression stockings is uncomfortable. The top rolls down and digs into my upper thigh, causing so much pain that I can’t walk more than a few steps.” [Participant 13, 7-year lymphedema].

#### 3.2.2. Theme 2: perceived and expected social support

##### 3.2.2.1. Categorie 1: family involvement in daily life management

Findings from the external behaviors within the daily life behavior dimension indicate that most family members provide as much assistance as possible after the patient is discharged, offering emotional support and actively participating in daily management. “My sister-in-law also has leg swelling after a hysterectomy. Sometimes when we chat, I feel much more relaxed.” [Participant 1, 2-year lymphedema]; “My husband has always been the 1 helping me wrap the bandage. My family’s super sweet to me. I don’t have to lift a finger for housework, like doing the laundry or cooking. Every day, I just eat, have a blast, and then eat some more. It’s so laid-back and stress-free.” [Participant 13, 7-year lymphedema].

##### 3.2.2.2. Categorie 2: expectation for telemedicine support

Several participants expressed a desire to receive remote professional support, consultation services, and medical guidance while recovering at home. “I wish a professional doctor could guide me on massage drainage techniques. When I follow online videos, I have no idea if I’m doing it correctly: if I do it wrong, it might be worse than not doing it at all.” [Participant 2, 3-year lymphedema]; “Over time, I forget which exercises to do and whether I’m doing them right. Having someone provide online guidance would definitely help.” [Participant 4, 5-year lymphedema]; “The hospital should provide more videos on post-surgical swelling management. It would be much more convenient for us patients to follow along.” [Participant 13, 7-year lymphedema]; “You need to recommend the right compression stockings for me… It would be great if follow-ups could be done online.” [Participant 16, 1-year lymphedema].

#### 3.2.3. Theme 3: inadequate ability to manage internal stress

##### 3.2.3.1. Categorie 1: fear of worsening swelling

Many participants expressed significant psychological stress and uncertainty, fearing that their lymphedema might worsen, which led to anxiety, nervousness, and other negative emotions. “I keep wondering whether the surgery will actually work in the long run.” [Participant 2, 3-year lymphedema]; “I often think: will I recover if I persist for 6 months? My whole family is concerned about it. After going through so much for the surgery to show results, I really hope it doesn’t fail.” [Participant 7, 11-year lymphedema]; “Are there any cases of complete recovery? I don’t know what the best possible outcome looks like after surgery.” [Participant 8, 13-year lymphedema].

##### 3.2.3.2. Categorie 2: high internal stress

“I used to be someone who couldn’t sit still, but now I feel so anxious. It’s frustrating that I can’t do anything because of my condition. When the stress builds up, I can’t sleep and my mind just keeps racing.” [Participant 1, 2-year lymphedema]; “When I take off the bandages to shower and see that my legs are different colors, I get really nervous. All I want to do is finish quickly and elevate my leg in bed.” [Participant 4, 5-year lymphedema]. “I keep hoping it will get better, but as each day passes without much change, I start feeling restless. I keep telling myself that it’s not time yet.” [Participant 6, 19-year lymphedema].

##### 3.2.3.3. Categorie 3: illness-related stigma

“Even when I go for a walk, I worry that people will notice. My leg looks terrible: I’m afraid of being laughed at, so I don’t dare stay outside for too long.” [Participant 2, 3-year lymphedema]; “The bandage on my upper arm makes it look really bulky. When I go out, my appearance suffers: people can immediately tell I have a medical condition.” [Participant 5, 5-year lymphedema]; “I used to think that when I got older, I couldn’t just rely on my children: I needed to find a partner. But with my leg like this, I don’t think I can anymore.” [Participant 6, 19-year lymphedema].

### 3.3. Self-concept behavior dimension

#### 3.3.1. Theme 1: accepting the disease and oneself

##### 3.3.1.1. Categorie 1: adapting to the disease and its consequences

“Before my leg swelled, I used to search every day about how long people survive with cervical cancer: it scared me so much. But now, I’ve come to terms with it. Living in the moment is what really matters. Right now, my focus is just on managing my leg properly.” [Participant 1, 2-year lymphedema]; “I’m so used to wrapping my legs every day. I just need to take good care of my swollen legs, and then I can live a normal life anyway. ” [Participant 2, 3-year lymphedema]; “I used to get so angry, wondering why I had to deal with cancer and then this condition on top of it. But now I think: at least I’m recovering, at least I can walk again. That’s already a win.” [Participant 8, 13-year lymphedema].

##### 3.3.1.2. Categorie 2: understanding the meaning of life

“Being able to live a healthy life is truly a blessing. Now, I just focus on working hard, living well, and enjoying my meals.” [Participant 8, 13-year lymphedema]; “I’m in a lymphedema support group, and whenever someone is feeling down, I always try to comfort them. Life is short: happiness is the most important thing.” [Participant 9, 4-year lymphedema]; “Looking back, I realize that all my past worries came from caring too much about others. It was exhausting. Now, I find that the more time I spend alone, the freer I feel. My mindset is in a really good place.” [Participant 11, 1-year lymphedema].

## 4. Discussion

Self-management is an effective approach for chronic disease management both domestically and internationally. Its core concept emphasizes the patient’s agency and self-responsibility, aiming to enhance the patient’s ability to manage their disease and achieve optimal health outcomes.^[21,22]^ Multiple randomized controlled trials have shown that good self-management can directly affect the recovery and quality of life of chronic disease patients, reducing the occurrence of adverse consequences.^[23–25]^ Reed, Granger, and Burton utilized the Chronic Disease Trajectory Framework to conduct in-depth interviews with patients suffering from various chronic diseases. They analyzed the characteristics of illness stages in these patients and created trajectory diagrams. This approach allowed for a clearer observation of the disease features and stage-specific needs of this patient population.^[26–28]^ This framework plays a significant role in the management of chronic disease patients by providing targeted nursing strategies and support tailored to different diseases. Researchers have used the Chronic Disease Trajectory Framework to categorize and track breast cancer patients, analyzing their disease stage characteristics and creating trajectory maps. This approach allows for a clearer observation of patients’ disease features and stage-specific needs, providing a basis for developing rational nursing strategies.

In this study, in-depth interviews and analyses were conducted with 16 patients with secondary lymphedema who had undergone LVA. Key findings were obtained. In terms of disease-related behaviors, patients had a significant lack of knowledge about postoperative rehabilitation, many misconceptions in self-management, difficulties in obtaining effective disease management information, and suboptimal self-management practices. In daily living behaviors, while compression therapy had some benefits, it also caused significant inconvenience to patients’ daily activities. Patients had limited ability to cope with psychological stress but expressed a strong need for social support, especially telemedicine support. In the dimension of self-concept behavior, most patients showed a positive side, gradually accepting the disease, adjusting their mindset, redefining the meaning of life, and actively participating in treatment and rehabilitation. These findings comprehensively present the complex situation of patients’ postoperative self-management and provide an important basis for developing targeted interventions.

### 4.1. Disease-related behavioral aspects: healthcare providers should prioritize post-LVA home self-management guidance

The investigation of home self-management in secondary lymphedema patients after LVA through the Chronic Illness Trajectory Model serves as the foundation and key for implementing precision rehabilitation nursing. Within the disease-related behavioral dimension, participants generally demonstrated incomplete understanding of postoperative rehabilitation knowledge, insufficient awareness of self-management importance, and evident cognitive misconceptions. Some patients exhibited discrepancies between self-management behaviors and cognition, lacked recognition of the importance of condition monitoring and follow-up and demonstrated limited experience in complication management. These cognitive limitations ultimately resulted in deficiencies in self-management skills and reduced compliance, a finding consistent with previous research identifying barriers in patient self-management processes.^[29]^ Consequently, improving the accessibility and dissemination of rehabilitation knowledge is crucial for enhancing patients’ self-management capabilities.

This study found that although patients received self-management education from healthcare professionals during hospitalization, similar to previous research,^[30]^ they often faced practical challenges during home rehabilitation, struggling to effectively recall and implement relevant measures, leading to omissions and oversights. Therefore, in clinical practice, healthcare providers could utilize follow-up software to effectively monitor patients’ daily care behaviors, such as through daily reminders or check-in mechanisms, to ensure timely execution of key measures including skin care, bandaging, manual lymphatic drainage, and functional exercises. Patients should be encouraged to upload photos or videos during and after procedures for remote therapist assessment and guidance, ensuring correct implementation of each care step. Concurrently, it is recommended that medical institutions establish specialized lymphedema rehabilitation clinics, enabling discharged patients to receive 15 to 21 days of complete decongestive therapy until they master standardized rehabilitation protocols. Additionally, these clinics should employ psychotherapists alongside certified lymphedema therapists to provide continuous psychological support during rehabilitation.

Participants generally reported low awareness of lymphedema in primary care institutions and community health service centers, coupled with non-authoritative and non-targeted online information, which increased difficulties in obtaining effective support and guidance. Therefore, enhancing lymphedema knowledge and disease management among primary healthcare providers may be a crucial pathway to promote effective patient self-management, contrasting with previous studies emphasizing patient education.^[31,32]^ Medical institutions should establish chronic disease trajectory management collaboratives for secondary lymphedema, building bridges between health education and self-management through discharge guidance, community health activities, and authoritative information resources. This approach will enhance public and professional awareness, ensure effective home rehabilitation support, and foster patients’ understanding and practice of disease knowledge and self-management skills, thereby activating their subjective initiative in disease management.^[33–35]^

The rapid development of internet technology has created new opportunities for self-management in lymphedema patients.^[36]^ A meta-analysis demonstrated that internet-based tools such as websites and social media software can effectively improve self-management behaviors in breast cancer patients and reduce lymphedema incidence.^[37]^ This study also found that most secondary lymphedema patients expect to obtain disease management information online and desire telemedicine support. Therefore, medical institutions should actively explore the integration of traditional healthcare with internet technology, improve the construction of specialized health information platforms, and establish telemedicine support systems.^[38]^ For example, developing a one-stop specialized health information platform integrating disease knowledge education, self-management guidance, doctor–patient communication, online consultations, and self-health monitoring. Clinical departments can utilize this platform to provide patients with online consultations, condition assessments, and treatment plan adjustments. Simultaneously, fully leverage WeChat and short video platforms to regularly publish lymphedema rehabilitation guidance information and encourage patient interaction.^[39]^

Furthermore, professional healthcare providers are crucial for lymphedema treatment. Studies indicate that the key to successful lymphedema treatment lies in care provided by specially trained and experienced nurses.^[40]^ Therefore, departments should cultivate 1 to 2 certified lymphedema specialist nurses.^[41]^ During hospitalization, these specialists should repeatedly emphasize postoperative LVA precautions to patients and families, and distribute illustrated health education manuals to enhance knowledge acquisition. Post-discharge, continuous professional support should be provided through remote consultations, regular follow-ups, rehabilitation guidance, and psychological support to help patients effectively manage their condition, resolve rehabilitation challenges, and improve treatment outcomes and quality of life.

### 4.2. Daily living behavioral aspects: enhancing psychosocial support for post-LVA patients

The internal behavioral dimension of daily life includes stress management and emotion regulation.^[42]^ This study showed that 40% of participants had significant clinical barriers in managing disease-related psychological stress and anxiety, which may stem from uncertainty about disease progression and loss of control over daily activities. This finding aligns with You et al conclusion that 58% of breast cancer patients with long-term home lymphedema care have anxiety disorders.^[43]^ Chronic psychological stress has been confirmed to exacerbate lymphatic stasis by increasing serum cortisol levels (β = 0.37, *P* < .01) through a TNF-α-mediated pathway,^[44,45]^ highlighting the necessity of integrating mental health support into comprehensive treatment plans to improve rehabilitation outcomes.^[46,47]^ Based on this, 2 evidence-based intervention strategies are prioritized in clinical practice: first, systematically incorporating licensed clinical psychologists into multidisciplinary care teams to provide personalized cognitive-behavioral therapy and group mindfulness training; second, implementing a family-centered structured care model through caregiver training programs to equip them with symptom monitoring skills and involve them in shared decision-making, thereby alleviating patients’ psychological stress and distress.

In the dimension of external daily life behaviors, this study revealed the significant impact of compression therapy on patients’ daily lives. Interviewees commonly reported that bandaging restricted limb mobility, making daily activities such as riding electric bicycles, climbing stairs, and doing housework difficult. Additionally, compression therapy affected urinary habits and sleep quality, exacerbating psychological distress. Studies confirm that proper bandaging techniques and graduated compression stockings are not only critical for achieving optimal therapeutic outcomes but also effectively alleviate discomfort from compression therapy and improve quality of life.^[48]^ Therefore, healthcare providers should tailor compression therapy plans to patients’ physiological conditions, activity levels, psychological states, and tolerance. When prescribing graduated compression stockings, clinicians must consider edema severity (ISL staging), limb dimensions, and individual pressure tolerance thresholds. Professionally guided precise measurement and fitting are essential to balance therapeutic efficacy and patient comfort.

### 4.3. Self-concept behavioral dimension: guiding post-LVA patients to develop positive self-concepts

Self-concept directly influences depressive symptoms and life satisfaction, with positive self-perception enhancing patients’ ability to develop effective disease coping strategies and improve self-management capabilities.^[42]^ The study found that participants demonstrated significant adaptability and positive attitudes in their self-concept behavioral dimension, whether transitioning from fear of disease prognosis to focusing on current life or shifting from illness-related anger to positive expectations for treatment outcomes. This psychological adaptation may stem from participants’ reevaluation of life’s meaning and value after facing dual challenges of cancer and lymphedema. Most interviewees reported receiving increased familial care and support in daily life post-discharge, enabling them to readjust mentally, reintegrate into daily activities, and maintain emotional stability. This psychological adaptation and reconstruction of self-identity not only enhanced their engagement in treatment and rehabilitation but also improved mental health and social functioning.

Previous studies have also shown that a positive self-concept is a significant predictor of health behaviors and exerts a substantial influence on them.^[49]^ Healthcare providers and caregivers should recognize that promoting positive transformations in patients’ self-concept is crucial for improving treatment outcomes and quality of life. This requires clinical teams to address patients’ psychological needs, including managing inferiority complex, depression, and body image changes caused by the disease.^[50,51]^ Family members should be encouraged to participate in patients’ daily disease management to enhance intra-family care and support, helping patients maintain a positive self-cognitive state. Additionally, patients can be encouraged to engage in peer community interactions to facilitate experience-sharing and mutual learning of self-management strategies, which helps sustain a positive self-concept collectively.

In summary, this study systematically explored the post-surgical home self-management experiences of patients with secondary lymphedema through 3 dimensions of the Chronic Illness Trajectory Model: illness-related behaviors, daily life behaviors, and self-concept behaviors. The results showed that some patients faced difficulties in accessing disease-related information, coping with daily activities, and managing the disease, indicating a need to improve their self-care abilities. This finding is consistent with the conclusions of Koczwara B and Chen J.^[52,53]^ De Vrieze T’s research revealed that the average annual cost of conservative treatment for lymphedema patients ranges from $2306 to $2574,^[54]^ and poor self-management further exacerbates the long-term physical and psychological challenges of the disease. Another study indicated that patients generally regard lymphedema as a persistent physical and psychological burden, which is consistent with the results of this study.^[55,56]^ Therefore, in-depth exploration of patients’ post-surgical home self-management experiences and identification of existing problems are of great significance for optimizing treatment outcomes and improving patients’ self-management abilities.^[57–59]^ The expert panel of the American Society of Breast Surgeons recommended that patients should establish long-term contact with professional lymphedema specialists to learn management strategies.^[60]^ Combined with this study, correct disease cognition, role adjustment, emotion management, application of coping strategies, and seeking professional support are the keys to improving patients’ self-management abilities.

## 5. Summary

This study systematically explored the post-discharge home self-management experiences of patients with secondary lymphedema from 3 dimensions of the Chronic Illness Trajectory Model: illness-related behaviors, daily life behaviors, and self-concept behaviors. The results revealed that patients commonly faced multidimensional challenges: insufficient rehabilitation knowledge, nonstandard self-management practices, and limited access to information at the illness management level, increasing the risk of complications; physical activity limitations, sleep disorders, and other discomforts caused by compression therapy at the daily life level, forming a “physiological-psychological” vicious cycle with psychological burdens; and although some patients gradually accepted the disease and reconstructed their life meanings at the self-concept level, 40% of participants experienced psychological issues such as anxiety and stigma.

This study indicated that patients’ self-management efficacy was comprehensively influenced by knowledge, skills, social support, and psychological status, while the existing care system had structural gaps in long-term post-surgical management, such as insufficient educational resources and weak primary healthcare support. Based on this, 3 intervention strategies were proposed: first, establishing a systematic rehabilitation education system to enhance patients’ disease management capabilities through healthcare provider training, standardized educational material development, and digital platform applications; second, implementing personalized compression therapy by customizing treatment plans according to individual characteristics, optimizing compression device selection, and providing assistive devices to reduce the negative impact of treatment on daily life; third, constructing a multidimensional psychological support network to alleviate psychological stress, eliminate stigma, and promote social integration through multidisciplinary team interventions, social support groups, and public awareness campaigns, providing an empirical basis for the construction of a “trajectory-oriented” intervention model.

This study has certain limitations. First, the sample size was small, with only 16 patients recruited through purposive sampling, all from a tertiary hospital in Shandong Province. Patients in different regions and healthcare settings may vary in disease perception, self-management abilities, and needs, potentially limiting the generalizability of the results to all patients with secondary lymphedema. Second, the study had a limited time span, focusing only on the short-term rehabilitation phase after discharge without tracking the long-term rehabilitation process. As time progresses, patients’ self-management experiences, needs, and disease progression may change, making it difficult to fully understand the entire rehabilitation cycle. Additionally, data collection relied primarily on patients’ subjective feedback, lacking objective data validation such as limb circumference measurements or lymphatic flow assessments, which may lead to inaccurate evaluations of patients’ self-management status.

Future research could be improved and expanded in several aspects: first, combining qualitative and quantitative research methods to further explore key factors influencing patients’ post-surgical home self-management (such as psychological traits, social support levels, and medical resource utilization) based on qualitative findings, and clarifying the impact and mechanisms of these factors through quantitative analysis to provide a more solid theoretical foundation for developing targeted interventions. Second, conducting long-term follow-up studies to observe the dynamic changes in patients’ self-management abilities, disease progression, and quality of life throughout the rehabilitation process, providing more forward-looking recommendations for long-term rehabilitation plans. Third, exploring the application and feasibility of new technologies in lymphedema rehabilitation, such as investigating the accuracy of wearable devices in monitoring limb swelling and movement data, and the effectiveness of smart rehabilitation aids in improving patients’ self-management efficiency and rehabilitation outcomes, to support innovation in rehabilitation technologies and enhance patients’ rehabilitation experiences.

Supplemental digital content “Appendix” is available for this article (https://links.lww.com/MD/P608).

## Author contributions

**Conceptualization:** Yinyin Guan, Hui Ju, Yuxia Fang.

**Data curation:** Yinyin Guan, Hui Ju, Zhenzhen Wan, Yuxia Fang.

**Formal analysis:** Hui Ju, Lanwei Xu.

**Investigation:** Yinyin Guan, Hui Ju, Lanwei Xu, Zhenzhen Wan, Lei Ge.

**Project administration:** Yuxia Fang.

**Resources:** Yinyin Guan.

**Software:** Lanwei Xu.

**Supervision:** Yuxia Fang.

**Validation:** Yuxia Fang.

**Visualization:** Lanwei Xu, Zhenzhen Wan.

**Writing – original draft:** Yinyin Guan, Hui Ju, Lei Ge

**Writing – review & editing:** Yuxia Fang

## Supplementary Material


