# Supplementary material for: A qualitative study on the post-discharge self-management experiences and needs of patients with secondary lymphedema
Source: Medicine (Baltimore). 2025 Aug 15;104(33):e43557. doi: 10.1097/MD.0000000000043557 (PMC12367003; doi:10.1097/MD.0000000000043557)
Supplement: Supplementary file 1 [file medi-104-e43557-s001.docx]

**Appendix：Interview Guide**

**Opening Question**

Can you tell me about your experience in managing your daily life? What insights and needs do you have?

**Main Questions**

① How much do you know about self-management at home after LVA surgery?

② How have you managed your condition after discharge?

③ What difficulties in daily life and work have you encountered during the process of self-management at home after surgery?

④ What support have your family and the outside world provided during your self-management at home after surgery? What kind of help do you expect to receive?

⑤ How has your mindset changed from the time of surgery to the period of self-management at home?
